# Fatty liver index as a predictor for incident type 2 diabetes in community-dwelling adults: longitudinal findings over 12 years

**DOI:** 10.1186/s12933-022-01642-1

**Published:** 2022-10-13

**Authors:** In-Ho Seo, Hye Sun Lee, Yong-Jae Lee

**Affiliations:** 1 Department of Family Medicine, Yonsei University College of Medicine, Gangnam Severance Hospital, 211 Eonju-ro, Gangnam-gu, Seoul, 06273 Korea; 2 Biostatistics Collaboration Unit, Yonsei University College of Medicine, Seoul, Korea

**Keywords:** Fatty liver index, Type 2 diabetes, Prospective cohort study, Prediction

## Abstract

**Background:**

Diagnosing fatty liver and identifying disease status are important for fatty liver related-diseases prevention. The fatty liver index (FLI), which can be easily available in clinical practice, can be very useful for managing fatty liver and preventing related diseases.

No large-scale and long-term follow-up prospective studies have investigated the relationship between FLI and incident type 2 diabetes (T2DM) independent of baseline insulin resistance status. Therefore, this study aimed to evaluate the association between FLI and incident T2DM and to determine whether FLI could be used as an indicator of T2DM using a large-sample, community-based Korean cohort over 12 years.

**Methods:**

Among the 10,030 total participants, 7,777 (3,676 men and 4,101 women) without diabetes were selected from the Korean Genome and Epidemiology Study (KoGES). FLI grade, which ranged from 0 to 100, was categorized into three groups: low, FLI (< 30); intermediate, FLI (30–59); and high, FLI (≥ 60). The hazard ratios (HRs) with 95% confidence intervals (CIs) for incident T2DM were calculated using multivariate Cox proportional hazards regression models after adjusting for potentially confounding variables.

**Results:**

In total, 1,490 individuals (19.2%) developed T2DM during follow-up. Compared to the reference FLI (< 30), the HRs of incident T2DM for the FLI (30–59), and FLI (≥ 60) increased after adjusting for potentially confounding variables, including the HOMA-IR marker.

**Conclusions:**

FLI grade at baseline could be a future indicator of T2DM even when prior glucose or insulin (HOMA-IR) levels are normal.

## Background

Type 2 diabetes (T2DM) has become a significant cause of mortality and comorbidity in cardiovascular disease and several types of cancer. In 2014, the global prevalence of T2DM was 422 million patients, which is expected to double by 2030–2045 [1]. Similar to the global pattern, the prevalence of T2DM in South Korea increased rapidly from 8.6% in 2001 to 13.7% in 2016 [2]. In addition, T2DM has caused many socioeconomic problems as well as individual economic problems. Socioeconomic status is an essential factor in diabetes-related policies, and several studies showed that the incidence of T2DM is inversely correlated with education and income levels [3, 4]. The cost of medical care for T2DM also continues to rise. The International Diabetes Federation reported that global health expenditures from diabetes were USD 966 billion in 2021 and this was expected to rise to USD 1,028 billion by 2030 and USD 1,054 billion by 2045 [5]. In the United States, 57% of the average medical expenditure is due to diabetes, and people with diabetes spent 2.3 times more on medical expenditures than those without diabetes [6]. Furthermore, the indirect costs of T2DM add to the substantial socioeconomic problem; and thus, it is a critical public health concern to predict and prevent T2DM in at-risk populations [6].

One of the underlying mechanisms of T2DM is uncontrolled metabolism of glucose [7]. The liver plays an important role in controlling glucose in various pathways, including glycogenesis, glycogenolysis, glycolysis, and gluconeogenesis, and several studies have focused on the relationship between liver dysfunction and T2DM [8, 9]. In a previous study of 438,069 Canadian adults with T2DM, a hazard ratio of the incidence of pre-existing serious liver disease was 1.92 compared to those without T2DM [10]. Fatty liver disease, including non-alcoholic fatty liver disease (NAFLD), is common and associated with T2DM. The prevalence of NAFLD was 49–62% in T2DM patients, and 18–33% of patients with NAFLD had T2DM [11–14]. In the recent prospective study, patients with a higher histological stage of NAFLD have a higher incidence of T2DM and a higher risk of all-cause mortality [15]. More recently, experts from the European Liver Patients’ Association proposed NAFLD as metabolic dysfunction-associated fatty liver disease (MAFLD) by emphasizing the importance of metabolic risk factors [16]. MAFLD is known to increase all-cause mortality and the incidence risk of various cardiometabolic diseases as well as liver fibrosis and stiffness, independent of demographic and lifestyle factors [17].

Early evaluation and management of individuals at high risk of fatty liver is essential to prevent various diseases, including T2DM, and related mortality. Traditional diagnostic techniques for fatty liver include liver ultrasonography, magnetic resonance, and biopsy [18]. The gold standard of fatty liver diagnosis is liver biopsy [19]. However, liver biopsy is inefficient in many non-advanced fatty liver patients and has limitations such as sampling error, high cost, inter- and intra-observer variability, and the risk of severe complications and mortality [20]. More recently, the fatty liver index (FLI), which is used to diagnose and stage fatty liver disease, has been replacing liver biopsy for fatty liver diagnosis. FLI testing is readily available, as each component of FLI calculation is a routine measurement in clinical practice [19, 21]. Also, FLI has been suggested as an economical alternative for mass screening for fatty liver disease with reasonable sensitivity and specificity [18]. FLI can be obtained by a formula using triglycerides (TG), γ-glutamyl-transferase (γ-GT), body mass index (BMI), and waist circumference (WC) [22]. Olubamwo et al*.* investigated the association between the incidence of T2DM and FLI. However, the study was limited to male participants, and the sample size was also insufficient [23]. Therefore, we prospectively examined the association between FLI and incident T2DM using a community-based Korean cohort over a 12 year period, including both sexes and a larger sample size.

## Methods

### Study population

The dataset used in this study (Ansan-Ansung cohort) was obtained after review and evaluation of our research plan by the Korea Centers for Disease Control and Prevention (http://www.cdc.go.kr/CDC/eng/main.jsp). This study used data from the Korean Genome and Epidemiology Study (KoGES), a longitudinal prospective cohort study conducted by the Korean Centers for Disease Control and Prevention (KCDC), to examine the prevalence of risk factors for chronic diseases in Korea. The KoGES consists of six large prospective cohort studies categorized into population-based and gene-environment model studies. All data in this study were derived from the KoGES Ansan-Ansung cohort, a population-based KoGES cohort study.

**Fig. 1:**
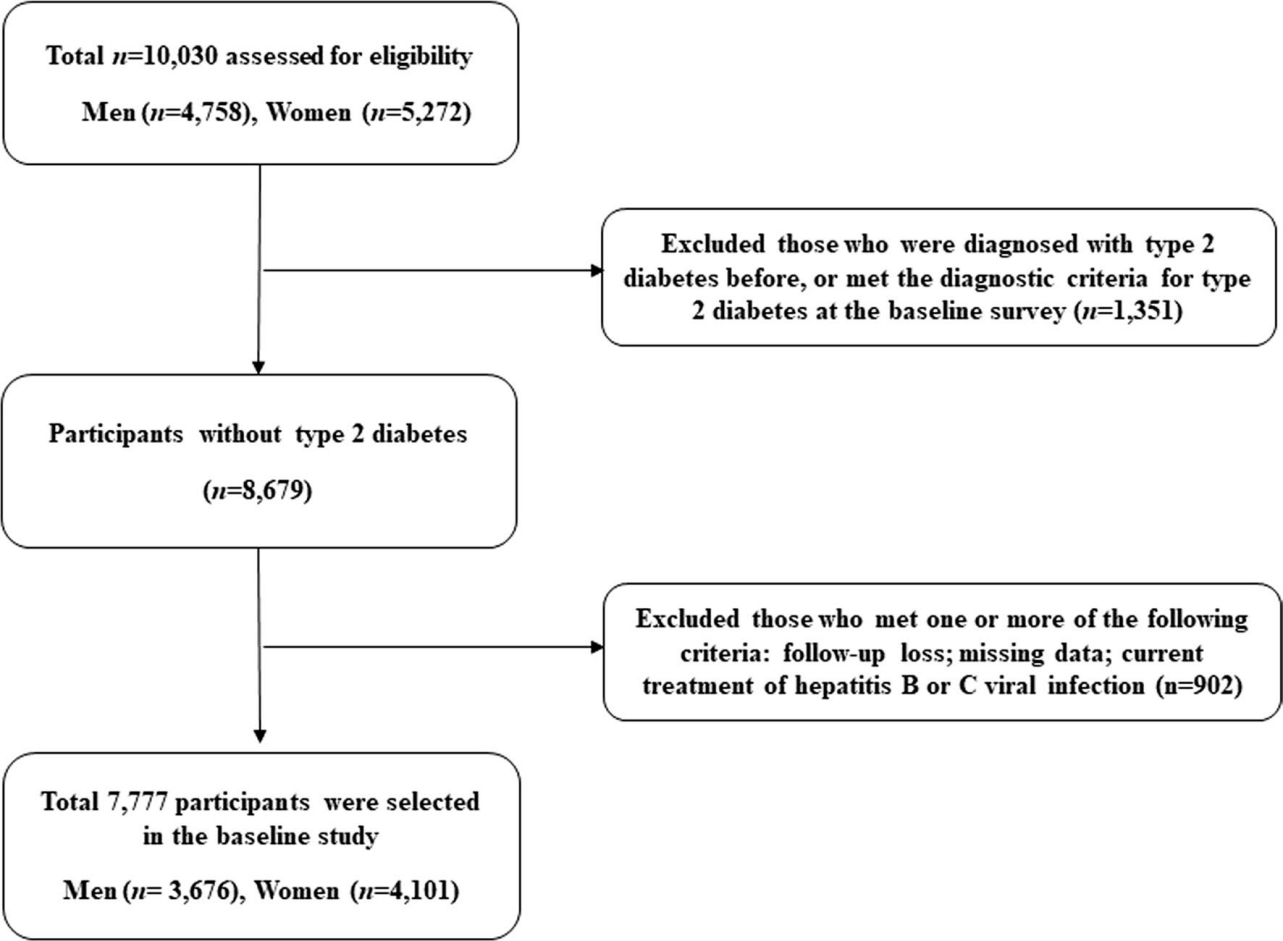
Flow chart of the study population selection

All participants voluntarily enrolled in the study, and all provided written informed consent. This study was conducted in accordance with the Declaration of Helsinki. The study protocol was approved by the Ethics Committee of the Korean Health and Genomic Study at the Korea National Institute of Health. Details of the KoGES, and its sampling methods have been reported elsewhere. The KoGES included men and women 40–69 years of age who lived in Ansan (urban area) or Ansung (rural area) during the baseline survey period from 2001–2002. The cohort was surveyed biennially until 2013–2014. Among the 10,030 participants in the baseline survey, 1,351 (13.5%) had been previously diagnosed with T2DM or satisfied the American Diabetes Association (ADA) diagnostic criteria for T2DM on the baseline survey and were excluded. Of the remaining participants, those who were lost to follow-up and who satisfied one or more of the following criteria also were excluded: missing data or current treatment of hepatitis B or C viral infection (n = 902). We defined missing data as the existence of any missing values among the covariates required for analysis. After these exclusions, 7,777 participants (3,676 men and 4,101 women) were included in the final analysis. A flow chart of the selection process is shown in Fig. 1.

### Study definitions and outcomes

Fasting plasma glucose, glycosylated hemoglobin (HbA1c), and post 2 h plasma glucose levels after a 75-g oral glucose tolerance test (OGTT) were evaluated biennially in all participants until 2013–2014. Based on the ADA criteria [24], new-onset T2DM was defined as any of the following: a fasting plasma glucose level  ≥ 126 mg/dL, a plasma glucose level  ≥ 200 mg/dL at 2 h after a 75-g OGTT, an HbA1c  ≥ 6.5%, or current treatment with oral anti-diabetic medications or insulin therapy. The formula for homeostasis model assessment of insulin resistance (HOMA-IR) was as follows: [fasting glucose (mg/dL) * fasting insulin (μIU/mL)/405]. FLI was calculated based on measurements of TG, γ-GT, BMI, and WC using the following formula: ${\text{FLI}}\, = \,\left( {{\text{e}}^{{0.{953} \times {\text{ln }}\left( {{\text{TG}}} \right) + 0.{139} \times {\text{BMI}} + 0.{178} \times {\text{ln }}(\gamma - {\text{GT}}) + 0.0{53} \times {\text{WC }} - { 15}.{745}}} } \right)/\left( {{1}\, + \,{\text{e}}^{{0.{953} \times {\text{ln }}\left( {{\text{TG}}} \right) + 0.{139} \times {\text{BMI}} + 0.{718} \times {\text{ln }}(\gamma - {\text{GT}}) + 0.0{53} \times {\text{WC }} - { 15}.{745}}} } \right)\, \times \,{1}00$

where TG is in mg/dL, γ-GT is in U/L, WC in cm and BMI in kg/m^2^ [22].

### Measurement of anthropometric and biochemical parameters

Participants regularly attended community clinics for anthropometric measurements and biochemical tests performed by trained healthcare providers. Participant height and body weight were measured to the nearest 0.1 cm and 0.1 kg, respectively, while subjects wore light indoor clothing without shoes. Body weight, height, and body composition was assessed using multi-frequency bioelectrical impedance analysis (BIA; InBody 3.0, Biospace, Seoul, Korea). Smoking status, drinking behavior, and physical activity levels were obtained from self-reported questionnaires that all study participants completed during the interview period. Smoking status was divided into three categories: current smokers, ex-smokers, and never smokers. We categorized alcohol drinking status as current drinker or non-drinker. Regular exercise was defined as moderate-intensity physical exercise at least three times a week. We defined one episode of exercise as any physical activity that lasted for at least 30 min. Participant family history included first-degree relatives. The systolic and diastolic blood pressure values were assessed three times in the right upper arm using a standard mercury sphygmomanometer (Baumanometer, Baum, Copiague, NY, USA), and the mean of the second and third blood pressure readings was used for analysis.

Biochemical parameters of fasting serum glucose, HbA1c, 60 min OGTT, 120 min serum glucose, and lipid levels (total cholesterol, TG, and HDL-C) were measured enzymatically using a 747 Chemistry Analyzer (Hitachi 7600, Tokyo, Japan). The HbA1c level was assessed using high-performance liquid chromatography (VARIANT II; Bio-Rad Laboratories, Hercules, CA). Plasma insulin concentration level was determined using a radioimmunoassay (LINCO kit, St. Charles, MO, USA).

### Statistical analysis

FLI grade, which ranged from 0 to 100, was categorized into three groups: low, FLI (< 30); intermediate, FLI (30–59); and high, FLI (≥ 60). Depending on the normality of the distributions of continuous variables, the baseline characteristics of the study population according to FLI group were compared using one-way analysis of variance or the Kruskal–Wallis test. The chi-square test was used to compare categorical variables. Continuous data are presented as mean ± standard deviation (SD) or median interquartile range (IQR). Categorical data are shown as frequency. The low FLI group was defined as the reference group. The hazard ratio (HR) with 95% confidence interval (CI) for incident T2DM was calculated using multivariate Cox proportional hazards regression models after adjusting for potentially confounding variables. The HRs with 95% CIs for incident T2DM were calculated using multivariate Cox proportional hazards regression models after adjusting for potentially confounding variables. The cumulative incidence of T2DM was represented using a Kaplan–Meier curve. Log-rank tests were conducted to determine the differences in the cumulative incidence of T2DM among the groups. All analyses were conducted using SAS 9.4 software (SAS Institute Inc., Cary, NC, USA). All statistical tests were two-sided, and statistical significance was defined as *p* < 0.05.

## Results

The baseline characteristics of 7,777 participants without diabetes at baseline are presented in Table 1 according to FLI grade. Variables related with cardiometabolic status increased as FLI grade increased. The mean values of BMI, WC, systolic and diastolic blood pressure, mean arterial pressure, and fasting plasma glucose were higher in the high FLI grade than in the low FLI grade. Also, the median values of insulin and HOMA-IR, which is related with insulin resistance, significantly increased with increasing FLI grade [insulin: mean FLI (< 30), 6.4; FLI (30–59), 7.6; and FLI (≥ 60), 8.6; HOMA-IR: mean FLI (< 30), 1.29; FLI (30–59), 1.55; and FLI (≥ 60), 1.84]. Several variables indicating hepatic function also increased with FLI. AST and ALT levels in FLI (≥ 60) were higher than levels in FLI (< 30) and FLI (30–59) [AST: mean FLI (< 30), 25; FLI (30–59), 27; and FLI (≥ 60), 32; ALT mean: FLI (< 30), 19; FLI (30–59), 26; and FLI (≥ 60), 36]. γ-GT also showed the same pattern [mean FLI (< 30), 13; FLI (30–59), 26; and FLI (≥ 60), 54]. In addition, the proportions of current smoking, alcohol drinking, and unfavorable lifestyle habits increased with FLI grade.

**Table 1:** Baseline characteristics of the study population according to the fatty liver index

	Total	FLI (< 30)	FLI (30–59)	FLI (≥ 60)	*p*-value
N	7777	4565	2103	1109	
Male (%)	47.3	37.6	55.7	71.2	< 0.001
Age (year)	51.7 (8.8)	51.3 (8.9)	52.7 (8.7)	51.4 (8.4)	< 0.001
Body mass index (kg/m^2^)	24.4 (3.1)	23.0 (2.4)	25.9 (2.4)	27.7 (3.0)	< 0.001
Waist circumference (cm)	82.2 (8.7)	77.5 (6.7)	86.9 (5.8)	92.3 (6.9)	< 0.001
Systolic blood pressure (mmHg)	120.5 (17.9)	117.1 (17.6)	124.2 (17.1)	127.5 (17.4)	< 0.001
Diastolic blood pressure (mmHg)	79.9 (11.3)	77.3 (10.9)	82.7 (10.7)	85.5 (10.9)	< 0.001
Mean arterial pressure (mmHg)	93.5 (12.9)	90.6 (12.6)	96.5 (12.2)	99.5 (12.3)	< 0.001
Fasting plasma glucose (mg/dL)	82.7 (8.5)	81.3 (7.9)	83.9 (8.7)	86.1 (9.3)	< 0.001
Insulin ((μU/mL)	6.9 (5.1–9.5)	6.4 (4.9–8.5)	7.6 (5.5–0.1)	8.6 (6.1–11.3)	< 0.001
HOMA-IR	1.40 (1.03–1.93)	1.29 (0.97–1.72)	1.55 (1.12–2.10)	1.84 (1.25–2.48)	< 0.001
Total cholesterol (mg/dL)	189.6 (34.1)	183.8 (32.2)	194.8 (33.6)	203.6 (36.9)	< 0.001
Triglycerides (mg/dL)	131 (98–183)	108 (86–137)	164 (129–211)	235 (179–315)	< 0.001
HDL-cholesterol (mg/dL)	4.4 (10.0)	47.1 (10.1)	42.5 (9.2)	40.7 (8.8)	< 0.001
Aspartate aminotransferase (U/L)	26 (23–31)	25 (22–29)	27 (24–32)	32 (26–39)	< 0.001
Alanine aminotransferase (U/L)	22 (17–30)	19 (16–25)	26 (20–33)	36 (27–50)	< 0.001
γ-glutamyltransferase (U/L)	18 (12–33)	13 (10–19)	26 (16–42)	54 (31–92)	< 0.001
Current smoking (%)	25.1	20.4	28.4	37.8	< 0.001
Alcohol drinking (%)^a^	48.0	41.9	51.9	65.7	< 0.001
Regular exercise (%)^b^	25.3	25.8	34.8	23.8	0.276
Family history of diabetes (%)	10.2	10.3	9.5	10.6	0.528

Data are expressed as the mean (SD), median (interquartile range) or percentage. P values were calculated using 1-way ANOVA test, Kruskal–Wallis test and chi-square test

^a^Alcohol drinking  ≥ two times/week

^b^Regular exercise  ≥ three times/week

**Fig. 2:**
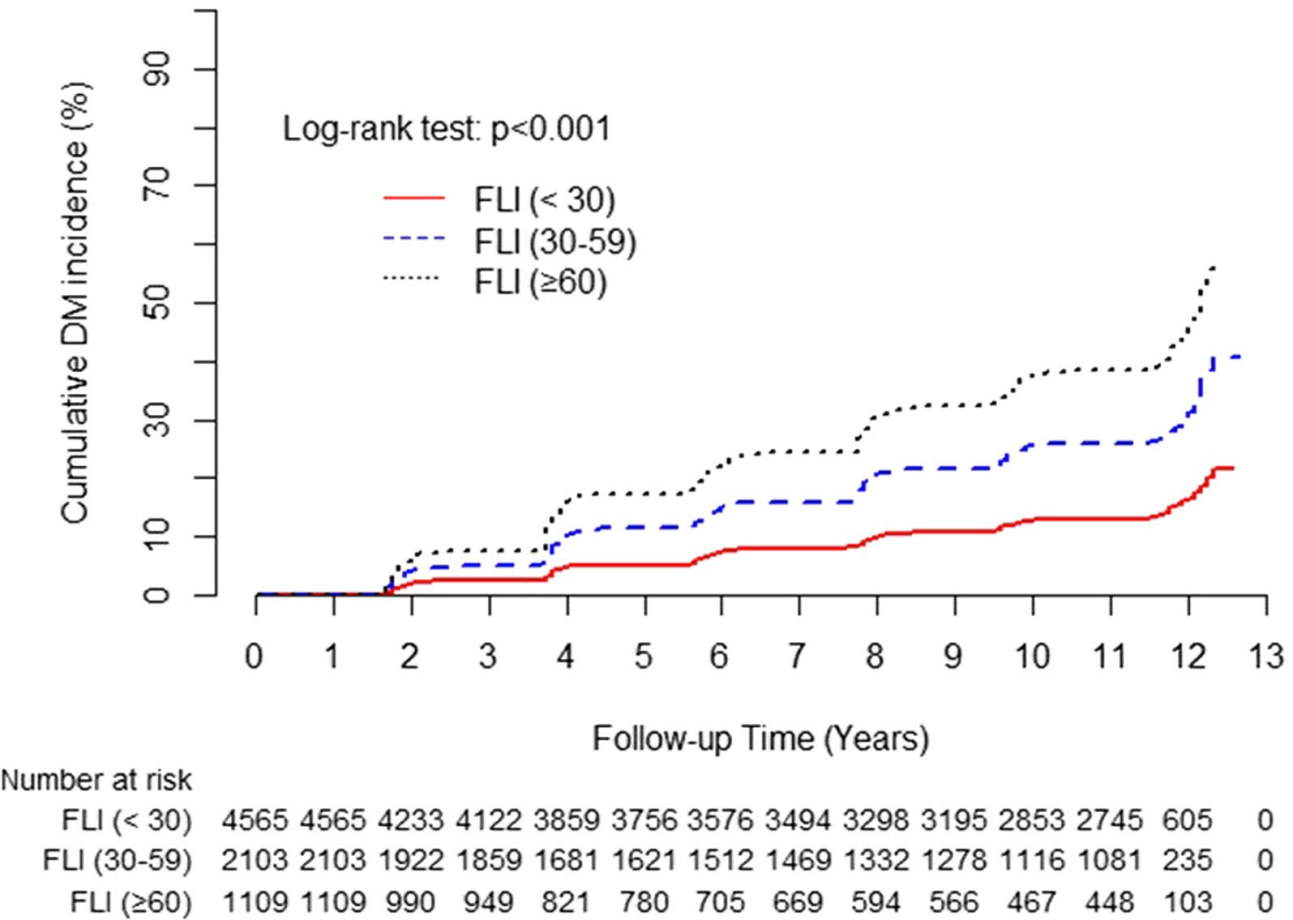
Cumulative incidence of T2DM according to FLI grade

Table 2 shows the incidence of T2DM during the 12 years of follow-up. During the follow-up period, the incidence rate of diabetes was calculated biennially. Of the 7,777 participants, 1,490 were newly diagnosed with T2DM during this 12 year follow-up period. The incidence rate per 2 years over the 12 year follow-up period was highest from 2005–2006 at 5.1 and lowest in 2013–2014 at 3.0. Figure 2 shows the cumulative incidence of T2DM according to FLI stage as a Kaplan–Meier curve. The cumulative incidence of T2DM over 12 years significantly increased as FLI grade increased (log-rank test *p* < 0.001).

**Table 2:** Incidence of type 2 diabetes during the follow-up study

Year range	Follow-up	n	Incidence cases (n)	Incidence rate over 2 years
2001–2002	Baseline	7777		
2003–2004	2 years	7285	284	3.9
2005–2006	4 years	6511	333	5.1
2007–2008	6 years	5841	257	4.4
2009–2010	8 years	5826	266	4.6
2011–2012	10 years	5509	193	3.5
2013–2014	12 years	5228	157	3.0

Further analyses to predict T2DM according to FLI stage were performed using multivariate Cox proportional hazards regression (Table 3). In Model 1, the HRs were calculated after adjusting for age, sex, WC, alcohol intake, and physical activity. In Model 2, mean arterial pressure and family history of diabetes were added in the analysis. Model 3 analysis including HOMA-IR was performed to consider insulin resistance. Compared to the reference FLI (< 30), the HR of incident T2DM for FLI (30–59) and FLI (≥ 60) increased in a FLI grade-dependent manner. The HR of T2DM incidence for FLI (≥ 60) was 3.41 (95% CI 2.98–3.90) in Model I, 3.13 (95% CI 2.72–3.59) in Model 2, and 2.98 (95% CI 2.58–3.43) in Model 3. The HRs in FLI (≥ 60) were significantly higher than HRs in the reference FLI (< 30) and in FLI (30–59) groups, independent of confounding variables, including HOMA-IR.

**Table 3:** Hazard ratios and 95% confidence intervals for incident type 2 diabetes risk according to fatty liver index

Fatty liver index (FLI)			
	FLI (< 30)	FLI (30–59)	FLI (≥ 60)
n	4565	2103	1109
New cases of diabetes (n)	579	515	713
Mean follow-up (years)	9.4 ± 3.5	8.8 ± 3.7	8.0 ± 3.8
Person-years of follow-up	42,926	18,444	8,856
Incidence rate/1000 person-years	13.5	27.9	80.5
Model 1	1.00 (reference)	2.04 (1.80–2.30)	3.41 (2.98–3.90)
Model 2	1.00 (reference)	1.94 (1.71–2.19)	3.13 (2.72–3.59)
Model 3	1.00 (reference)	1.89 (1.66–2.14)	2.98 (2.58–3.43)

Model 1: adjusted for age, sex, waist circumference, alcohol intake, and physical activity

Model 2: adjusted for age, sex, waist circumference, alcohol intake, and physical activity, mean arterial pressure and family history of diabetes

Model 3: adjusted for age, sex, waist circumference, alcohol intake, and physical activity, mean arterial pressure and family history of diabetes, and HOMA-IR

## Discussion

In this large, community-based, 12 year prospective cohort study, FLI was positively related to incident T2DM independent of baseline insulin resistance (measured as HOMA-IR) after adjusting for potential confounding variables. In addition, one advantage of the present study was that our results could be applied to participants with high FLI alone as well as those with MAFLD. The concept of MAFLD is independent of alcohol consumption amount and includes both alcoholic fatty liver disease and NAFLD [25]. Our study showed that FLI was associated with T2DM independently of the parameters required to diagnose MAFLD and also independently of alcohol drinking. The association between FLI and new-onset T2DM showed a pattern consistent with previous studies. Carla et al*.* showed that FLI can be a prediction marker for conversion to T2DM among those with pre-diabetes [26]. The incidence rates of T2DM in the FLI (≥ 60) group compared to other FLI groups were significantly different after adjustment for confounding factors (HR = 6.879, 95% CI 5.873–8.057 for men, and HR = 5.806, 95% CI 4.863–6.932 for women). Similarly, Franch-Nadal et al. suggested that FLI is a predictor of incident diabetes in patients with prediabetes [27]. However, these studies focused on men and women with a status of pre-diabetes. These limitations are difficult to extend to the general population. García-Escobar et al. performed baseline analysis of normoglycemia participants to determine the incidence of T2DM after adjusting for base model confounding factors of age, sex, fasting glucose, and family history of T2DM [28]. Additional analyses were performed after adjusting for limited confounding factors. In the base model, incidence rate ratio (RR) of FLI for new-onset T2DM was 4.10 (95% CI 1.48–11.33) in FLI (≥ 60) compared to the reference group FLI (< 30). Although the previous study is consistent with our findings as to the role of FLI in predicting T2DM, that study had several limitations. First, total subject size and new-onset T2DM subject size were small, and follow-up time was too short for accurate analyses (normoglycemic subjects, 1,619; new-onset T2DM, 37; and follow-up time, 7.5 years). Moreover, the authors did not include all confounding factors in analysis at once. Their analysis did not reveal the influence between confounding factors, which makes the role of FLI as an indicator unclear. Our results are consistent with a previous prospective study of 1,792 male participants in East Finland [23]. However, that study had limitations, such as small sample size and the lack of female participants. After addressing the limitations of the previous studies, the current study revealed a positive relationship between FLI and incidence risk of T2DM in both sexes regardless of baseline insulin resistance (such as HOMA-IR) in a long-term follow-up cohort study (12 years) with a large sample population.

The most plausible underlying mechanism for the relationship between FLI and T2DM is hepatic insulin resistance and hepatic inflammation. Insulin regulates lipids and glucose metabolism in the liver [29]. However, the accumulation of hepatic lipids causes altered lipoproteins, hepatotoxicity, oxidative stress inflammation, and increased lipogenesis and gluconeogenesis [30, 31]. As a result, hepatic glucose output increases while insulin clearance decreases [32]. Also, due to lipid accumulation, the increase in lipid oxidation leads to abnormalities in mitochondrial function, and generation of glycerol and long-chain fatty acids in adipocytes increases [32]. These mechanisms are involved in insulin resistance, hepatic inflammation, and the resulting increased pro-inflammatory response. In addition, problems with lipid and glucose regulation caused by abnormal bile acid action and genetic polymorphisms affect fatty livers and T2DM [32]. This linked mechanism explains the relationship between FLI and T2DM.

There are several limitations that should be considered when interpreting the findings of the present study. The KoGES cohort data used for this study were collected from a large, prospective dataset. However, all participants came from the Korean population, indicating that our results might not be generalized to other populations. Also, the cohort data had the potential for selection bias as participation was voluntary and limited to specific geographic regions. Therefore, future research should involve multicenter studies including people of various ethnicities and geographic regions. Despite these potential limitations, our present study suggests the clinical importance of FLI as an indicator of T2DM in East Asian populations.

## Conclusions

In conclusion, a high FLI precedes and significantly predicts future development of T2DM among community-dwelling and middle-aged to older Korean adults. Given the increasing prevalence of T2DM worldwide, our findings have important implications in terms of a more accurate model to predict incident T2DM.

## Data Availability

The dataset used in this study (Ansan-Ansung cohort) is available from the Korea Centers for Disease Control and Prevention (http://www.cdc.go.kr/CDC/eng/main.jsp) upon request.

## Abbreviations

T2DM: Type 2 diabetes
FLI: Fatty liver index
KoGES: The Korean genome and epidemiology study
HRs: Hazard ratios
Cis: Confidence intervals
NAFLD: Non-alcoholic fatty liver disease
MAFLD: Metabolic dysfunction-associated fatty liver disease
TG: Triglycerides
γ-GT: γ-Glutamyl-transferase
BMI: Body mass index
WC: Waist circumference
KCDC: Korean Centers for Disease Control and Prevention
ADA: American Diabetes Association
HbA1c: Glycosylated hemoglobin
OGTT: Oral glucose tolerance test
HOMA-IR: Homeostasis model assessment of insulin resistance
SD: Standard deviation
IQR: Median interquartile range
